# Biofilm-Forming Methicillin-Resistant *Staphylococcus aureus* Survive in Kupffer Cells and Exhibit High Virulence in Mice

**DOI:** 10.3390/toxins8070198

**Published:** 2016-06-30

**Authors:** Takuto Oyama, Motoyasu Miyazaki, Michinobu Yoshimura, Tohru Takata, Hiroyuki Ohjimi, Shiro Jimi

**Affiliations:** 1 Department of Plastic, Reconstructive and Aesthetic Surgery, Faculty of Medicine, Fukuoka University, Fukuoka 814-0180, Japan; oyt1974@yahoo.co.jp (T.O.); ohjimi@fukuoka-u.ac.jp (H.O.); 2 Department of Pharmacy, Fukuoka University Chikushi Hospital, Chikusino 818-8502, Japan; karl-101@hotmail.co.jp; 3 Department of Medical Oncology, Hematology, and Infectious Diseases, Faculty of Medicine, Fukuoka University, Fukuoka 814-0180, Japan; myoshimura@fukuoka-u.ac.jp (M.Y.); takattol@cis.fukuoka-u.ac.jp (T.T.); 4 Central Laboratory for Pathology and Morphology, Department of Pathology, Faculty of Medicine, Fukuoka University, Fukuoka 814-0180, Japan

**Keywords:** MRSA, virulence, biofilm, intracellular persistence, Kupffer cells, mice

## Abstract

Although *Staphylococcus aureus* is part of the normal body flora, heavy usage of antibiotics has resulted in the emergence of methicillin-resistant strains (MRSA). MRSA can form biofilms and cause indwelling foreign body infections, bacteremia, soft tissue infections, endocarditis, and osteomyelitis. Using an in vitro assay, we screened 173 clinical blood isolates of MRSA and selected 20 high-biofilm formers (H-BF) and low-biofilm formers (L-BF). These were intravenously administered to mice and the general condition of mice, the distribution of bacteria, and biofilm in the liver, lung, spleen, and kidney were investigated. MRSA count was the highest in the liver, especially within Kupffer cells, which were positive for acid polysaccharides that are associated with intracellular biofilm. After 24 h, the general condition of the mice worsened significantly in the H-BF group. In the liver, bacterial deposition and aggregation and the biofilm-forming spot number were all significantly greater for H-BF group than for L-BF. CFU analysis revealed that bacteria in the H-BF group survived for long periods in the liver. These results indicate that the biofilm-forming ability of MRSA is a crucial factor for intracellular persistence, which could lead to chronic infections.

## 1. Introduction

*Staphylococcus aureus* is a human pathogen that causes a range of illnesses, from minor skin infections to life-threatening disease. Methicillin-resistant *S. aureus* (MRSA) is one of most detectable microbes in hospital-acquired infections, and causes device-associated infections, bacteremia, soft tissue infections, endocarditis, and osteomyelitis [1,2,3]. MRSA has emerged as a significant threat in both hospital and community environments [4]. With limited treatment options, MRSA infections are associated with high mortality [5].

MRSA forms biofilms not only on indwelling medical devices and implants but also in tissues. Bacterial biofilm is thought to be an important factor in chronic infections. Biofilms are mainly composed of extracellular polysaccharides (EPS) produced by bacteria, and they also contain proteins and extracellular DNA [6,7,8]. Many *S. aureus* genes are involved in biofilm formation, including *fnb, ica*, *agr*, and *sarA*. The extracellular polysaccharide adhesin PIA/PNAG, which is encoded by the *icaADBC* locus, is responsible for the biofilm-forming phenotype [9]. Although elimination of biofilm is an important issue in medical practice, no effective methods are available.

Bacterial invaders adopt sophisticated strategies against host antimicrobial immunity in vivo. *S. aureus* biofilm can attenuate host pro-inflammatory responses and escape macrophage phagocytosis by evasion of Toll-like receptors [10]. Another important mechanism is enhanced drug resistance, especially in the biofilm state, which is explained by the presence of persisters and/or decreased drug permeability [6,11,12]. However, how biofilms exhibit ten to a thousand times increase in drug resistance compared to that of planktonic bacteria remains unknown.

In a healthy host, invading bacteria are generally ingested by phagocytic cells including macrophages, monocytes and neutrophils [13] and then digested and killed by lysosomal enzymes. However, a hallmark of staphylococcal infection is frequent recurrence as the bacteria manipulate host immune responses [14]. Bacteria also implement another strategy for intracellular survival [15,16,17]: they hide like Trojan Horses inside the cell and may only become virulent in immune-compromised patients. These bacteria include not only common parasitic bacteria such as *Shigella*, *Salmonella*, and *Mycobacterium tuberculosis*, but also *S. aureus* [18].

An epidemiological study using *S. aureus* from long-lasting bovine mastitis showed that in vitro bacterial internalization in bovine mammary epithelial cells is related to accessory gene regulator (*agr*) group I, but not to *agr* group II or biofilm formation [19]. It has been reported that internalized *S. aureus* is able to replicate inside pulmonary epithelial cells and induce apoptosis [20]. However, this is arguably a disadvantage for the bacteria in losing an intracellular safety location, since in this location, they are able to escape many host defense mechanisms and shield themselves from extracellular antimicrobial antibiotics. Survival of *S. aureus* within the macrophage phagosome requires specific mechanisms against normal microbicidal reactions. Das et al. (2008) [21] showed that catalase and superoxide dismutase produced by *S. aureus* combat intracellular oxygen species, allowing *S. aureus* to persist within macrophages. Another mechanism of intracellular survival of *S. aureus* is the small colony variant (SCV) [22,23]. However, the precise intracellular mechanisms of *S. aureus* survival in vivo are still unclear.

In the present study, to clarify the relationship between biofilm-forming ability and intracellular bacterial survival, clinical isolates of MRSA were intravenously injected into mice. MRSA isolates were then divided into two groups, high and low, by their biofilm-forming ability in vitro. Bacterial tissue distribution, including intracellular localization, was examined. This in vivo study showed that biofilm is important for intracellular MRSA survival and virulence.

## 2. Results

### 2.1. MRSA Isolates and Genotypes

OJ-1, a previously reported biofilm-forming isolate that had been collected from a wound surface [24], was used as the control. In a study based on differences in biofilm-forming ability, 20 clinical MRSA isolates from blood specimens were selected and classified as the low-biofilm formers (L-BF; 10 isolates) and high-biofilm formers (H-BF; 10 isolates) (Table 1). Mean Crystal violet (CV) levels for biofilm-forming ability in the H-BF group were significantly higher than that in the L-BF group (0.126 ± 0.02 and 1.232 ± 0.208, respectively, *p* < 0.001). No considerable difference was found between the two groups in terms of genotype (i.e., *agr,* SCC*mec*, *ica*, and *fnbA*) involvement in MRSA virulence and biofilm formation (Table 1 and Table S1).

### 2.2. Detection of Extracellular Polysaccharides in Biofilm in the Liver

**Figure 1:**
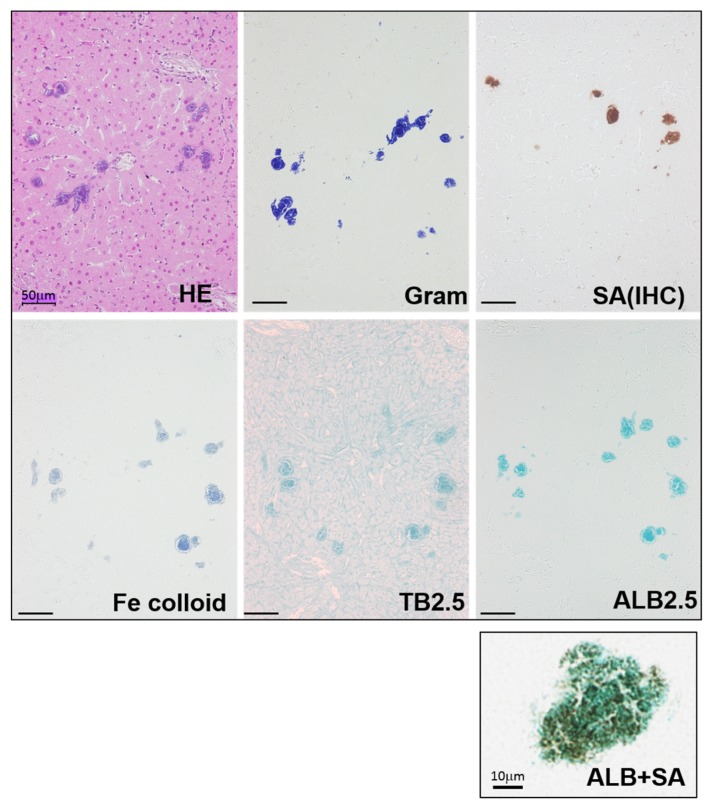
Detection of methicillin-resistant *S. aureu* (MRSA) colonies and their biofilm matrix in the liver.

Among the mice that received injections of MRSA isolates, some showed localized necrotic foci inside the liver. For the mice that showed bacterial clusters in the liver, serial sections were examined by different staining (Figure 1 and Figure S1). Colonies were present inside the necrotic foci, and their size ranged from 10 to 50 μm in diameter. Gram-positive bacterial nuclei were stained by hematoxylin and were also found to be positive for anti-*S. aureus* antibody. To detect biofilm matrixes, mucopolysaccharide staining was used. PAS staining for natural mucopolysaccharides failed to show any specific tissue localization. Meanwhile, when iron colloid staining, toluidine blue staining (pH 2.5), and Alcian blue staining (pH 2.5) (ALB) for acidic mucopolysaccharides were utilized, all of the staining was positive in the bacterial colonies. In addition, double staining using ALB staining and immunostaining of *S. aureus* was used to examine the relationship between the bacterial body and the extracellular polysaccharides. The results showed that circular and granular bacteria were assembled together and were surrounded by a biofilm matrix containing acidic mucopolysaccharides. Because of the detection capability of ALB staining, as well as its simplicity and broad histological utility, ALB staining was used for the following studies.

MRSA colonies had developed inside necrotic regions of the liver 24 h after bacterial injection, and were stained with HE staining, Gram staining, and immunostaining using an anti-*S. aureus* antibody. Acidic mucopolysaccharide staining, specifically iron colloid staining (Fe colloid), toluidine blue staining (pH 2.5) (TB), and Alcian blue staining (pH 2.5) (ALB), was also used. The bacterial cells and the extracellular polysaccharides were examined by a means of double staining using ALB and *S. aureus* antibody (ALB + SA). The surrounding matrix (blue) was positive for acidic mucopolysaccharides. Bars = 50 μm.

### 2.3. Time-Course of Bacterial Distribution and MRSA in Each Organ

**Figure 2:**
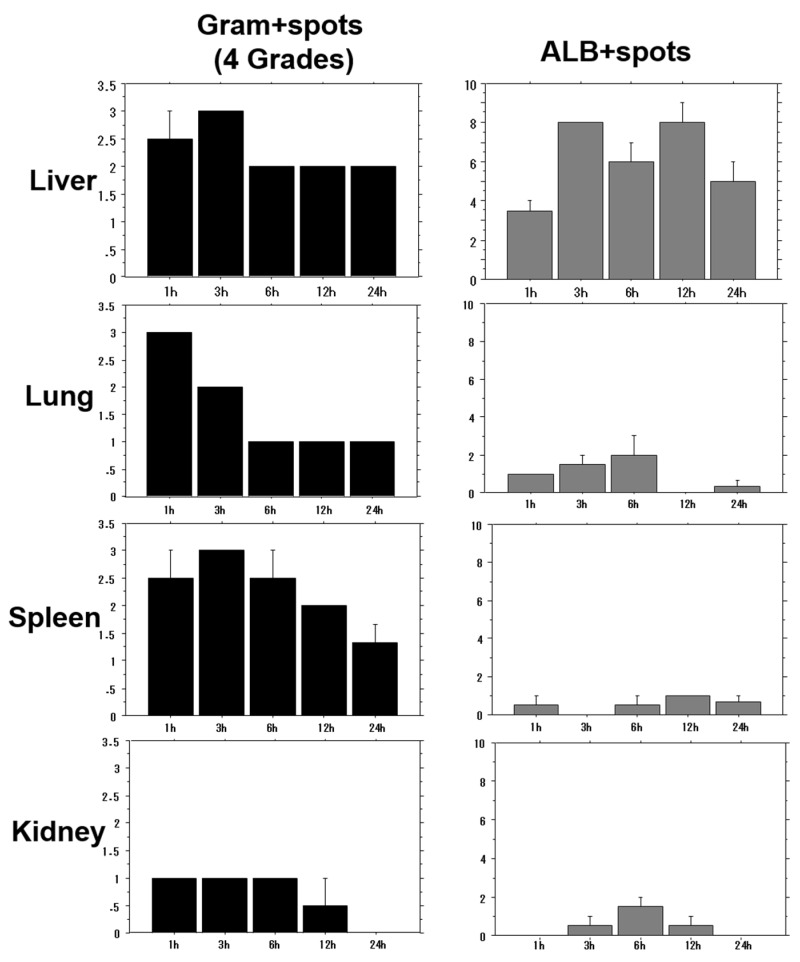
Time-course of bacterial density (**left**) and ALB spot number in different organs (**right**).

A bacterial solution of OJ-1 was intravenously injected into mice, and tissue distributions in the liver, lungs, spleen, and kidneys were examined at 1, 3, 6, 12, and 24 h. After tissue specimens were stained by Gram staining and ALB staining, the stained spots were morphometrically analyzed. The number of Gram-positive spots in the tissues (bacterial density) as well as the number of ALB-positive spots (extent of biofilm formation) was evaluated. Tissue bacterial density was lower in the kidneys than in the liver, lungs, and spleen (Figure 2). Although a time-course decrease in bacterial counts was found in all organs, persistence of residual bacteria was highest in the liver. Meanwhile, the number of ALB-positive spots was highest in the liver, showing that bacterial accumulation was greater in the liver than in other organs and was maintained even 24 h after bacterial injection. Similar results for tissue distribution, especially in the H-BF group, were also found in isolated bacteria from the blood.

The tissue distribution of OJ-1 in the liver, lung, spleen, and kidney was examined at 1, 3, 6, 12, and 24 h after intravenous bacterial injection. Tissue bacterial density was evaluated using Gram-stained specimens as a reference, as shown in Figures S2–S5. The number of ALB-positive spots was counted. Value: mean ± SEM.

**Figure 3:**
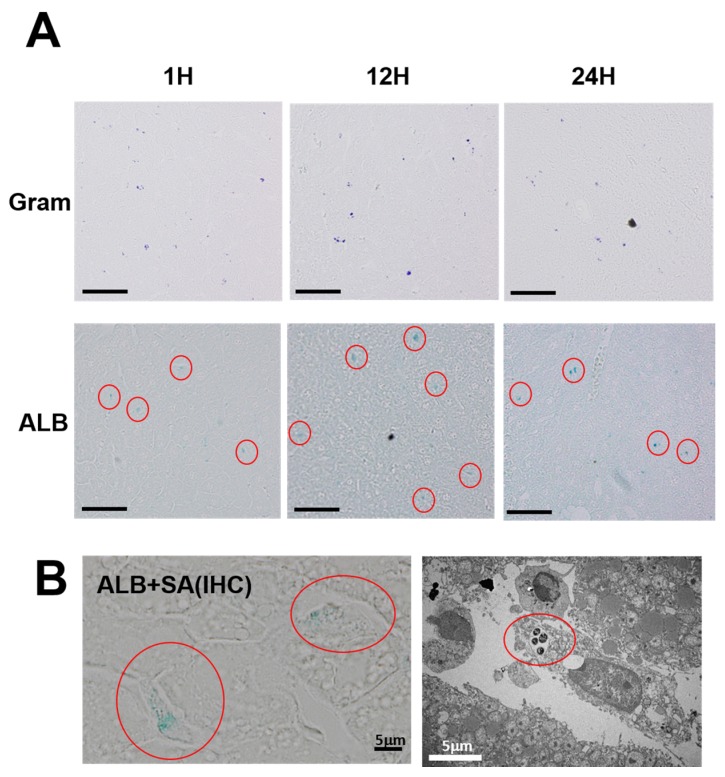
Intrahepatic distribution of bacteria and ALB-positive material. (**A**) Representative histological aspects of liver tissues with Gram staining and ALB staining (red circles) performed at 1, 12, and 24 h after intravenous injection of OJ-1. Bars = 50 μm; (**B**) Double staining of liver tissues using ALB and *S. aureus* antibody (SA) (**left**). ALB-positive spots were found inside the cells (red circles), and several units to dozens of *S. aureus* antibody-positive bacteria were visible at the same site. In electron microscopy (**right**), intracellular bacteria are localized in the vacuole, probably in the phagosome. Based on the cells' morphology and localization, they are Kupffer cells. Bars = 5 μm.

Both the Gram-positive and ALB-positive spots were distributed diffusely in the liver (Figure 3A). In order to investigate the details of the distribution profiles, *S. aureus* that had been subjected to double staining were observed by microscopy with strong magnification (Figure 3B, left). The images show the presence of several units to dozens of bacteria inside the ALB-positive spots, distributed primarily in Kupffer cells in the hepatic sinusoid. The electron microscopic picture also shows that intracellular bacteria are localized in the membrane-associated vacuole in a Kupffer cell (Figure 3B, right).

### 2.4. Time-Course of Intrahepatic Bacterial Accumulation

To examine the distribution pattern of OJ-1 in liver tissues, the number and surface area of spots at Gram-positive sites were analyzed. The aforementioned sites could be classified into two categories. One of the histograms showed a monomodal distribution of surface areas of 10 μm^2^ or less. Meanwhile, at sites containing spots with larger areas, another distribution was bimodal at the 10 μm^2^ spot, which showed intracellular accumulation/aggregation of bacteria.

**Figure 4:**
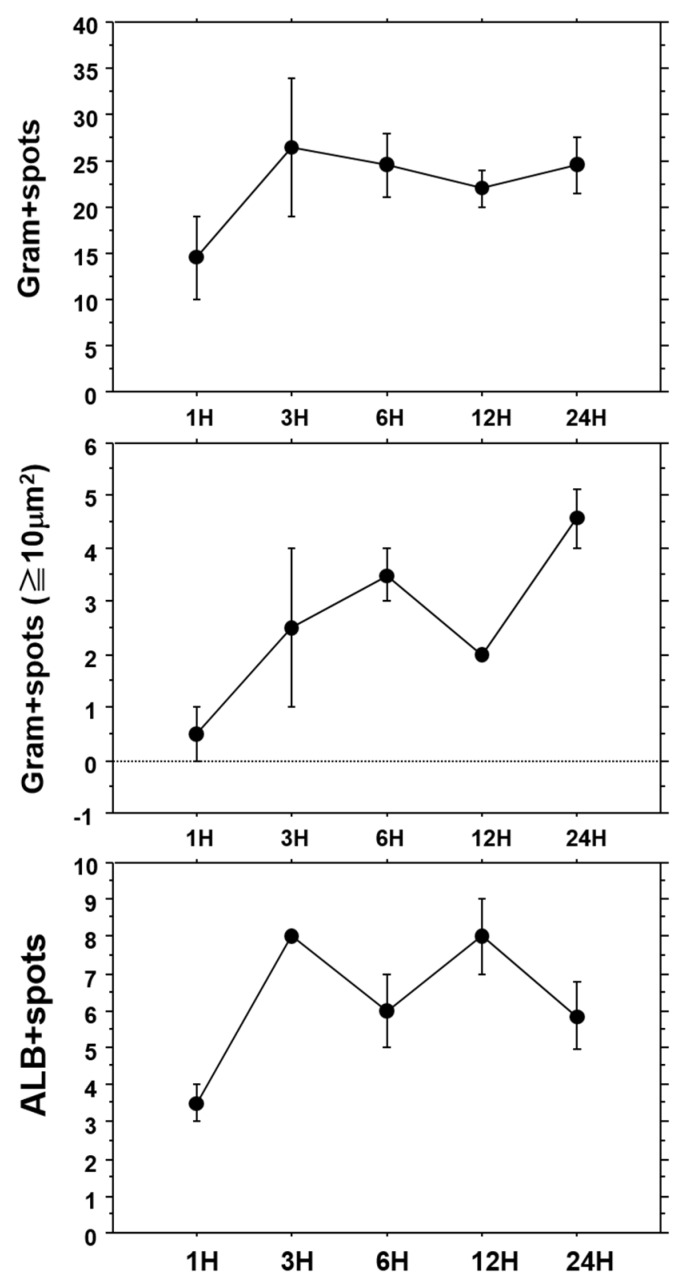
Time-dependent changes in tissue distribution patterns of Gram-positive and ALB-positive spots. This figure shows the time-course of the number of Gram-positive spots in the liver after injection of OJ-1 (**top**); number of Gram-positive spots with a surface area of 10 μm^2^ or greater (**center**); and number of ALB-positive spots (**bottom**). Value: mean ± SEM.

We examined the time-course of the number of Gram-positive spots in liver tissues after intravenous injection of OJ-1 (Figure 4, top), the number of Gram-positive spots with an area of 10 μm^2^ or greater (Figure 4, center), and the number of ALB-positive spots (Figure 4, bottom). Although the number of Gram-positive spots was small during the first hour after injection, it increased thereafter to a number that was maintained for 24 h. Meanwhile, the number of spots with aggregated bacteria with a surface area of 10 μm^2^ or greater increased with time during the 24 h of the study. Although the number of ALB-positive spots showed temporal variation, it stabilized after the first 3 h and did not decrease considerably. Similar results of time-dependent alteration in the three parameters were found in isolated bacteria from the blood. These results support that bacteria injected into the blood accumulate/aggregate in liver tissues, and are retained in liver tissues up to 24 h.

### 2.5. Relationship between Intrahepatic Bacterial Accumulation with the L-BF and H-BF Groups

**Figure 5:**
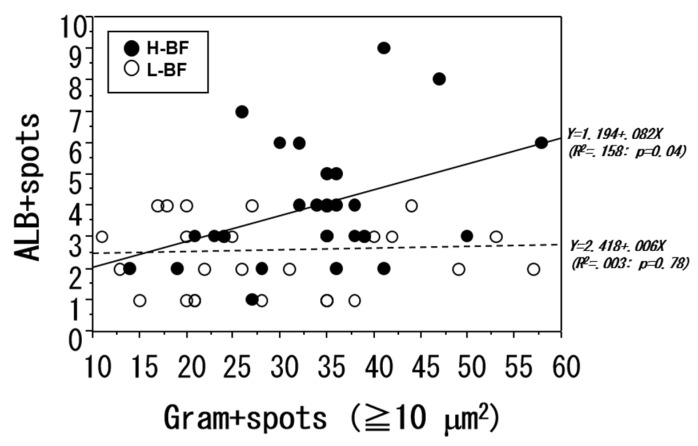
The relationship of intrahepatic bacterial accumulation with the L-BF and H-BF groups.

We examined the correlation between the number of bacterial aggregation spots measuring 10 μm^2^ or greater and the number of ALB-positive spots (Figure 5). No correlation was found between the two parameters. In addition, in the L-BF group, no significant correlation was found between the numbers of the two types of spots, whereas in the H-BF group, an increase in the number of bacterial aggregation spots was accompanied by a significant increase in the number of ALB-positive spots (*p* < 0.04).

In L-BF and H-BF groups, correlation analysis of the number of bacterial aggregation spots with 10 μm^2^ or greater and the number of ALB-positive spots in the liver after 24 h was performed. No correlation was found between the two parameters when all cases were examined together or when the L-BF group alone was examined. However, in the H-BF group, a significant primary correlation was found between the two parameters (*p* < 0.04).

### 2.6. Intrahepatic ALB-Positive Spot Numbers in L-BF and H-BF

**Figure 6:**
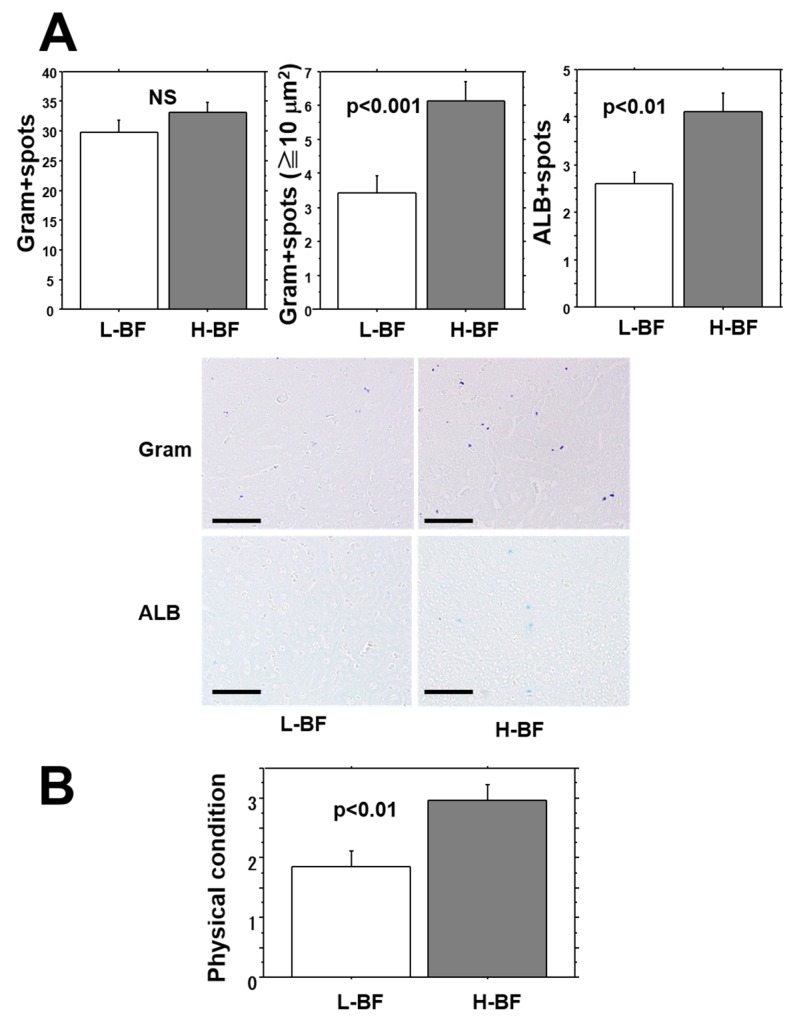
Intrahepatic bacterial aggregation and ALB-positive spot number 24 h after bacterial injection. (**A**) No difference was found in the number of bacterial spots in the liver between the groups, but the number of bacterial aggregates measuring 10 μm^2^ or larger was apparently greater in the H-BF group than in the L-BF group (*p* < 0.001). In addition, the number of ALB-positive spots showing biofilm formation was significantly higher in the H-BF group. The photos show representative samples stained by Gram and ALB. Bars = 50 μm; (**B**) General condition was assessed using a four-level evaluation. The general condition was significantly worse in the H-BF group than in the L-BF group (*p* < 0.01). Value: mean ± SEM.

Bacterial counts and ALB-positive spot numbers in the liver were examined 24 h after injection of isolates into the L-BF and H-BF groups (Figure 6A). While the number of bacterial spots in liver tissues showed no difference between the two groups, the mean number of bacterial aggregation spots measuring 10 μm^2^ or larger was markedly greater in the H-BF group than in the L-BF group (approximately 2 times greater) (*p* < 0.001). In addition, the mean number of ALB-positive spots indicative of bacterial accumulation was significantly higher in the H-BF group than in the L-BF group (*p* < 0.01).

### 2.7. General Condition of Mice after Injection of MRSA

At 24 h after bacterial injection, the general condition of the mice was compared using a four-level evaluation (Figure 6B). Worsening of the general condition was found to be significantly more severe in the H-BF group than in the L-BF group (*p* < 0.01). In addition, comparison based on fatalities among the mice revealed that 8 out of 30 mice (27%) died in the L-BF group and 16 out of 30 mice (53%) died in the H-BF group, showing that the fatality rate was 2 times higher in the H-BF group than in the L-BF group (*p* < 0.05).

### 2.8. Persistence of MRSA in the Liver

To avoid acute effects after bacterial injection, 1/2 diluted bacterial solution was utilized. At 24 h after injection, physical condition was not worsen, and bacteremia was virtually absent in both of the groups. The number of viable bacteria (log-CFU) in the liver was 2.8 ± 0.3/mg in L-BF and 3.8 ± 1.4/mg in L-BD and H-BF, respectively (*p* < 0.001).

**Figure 7:**
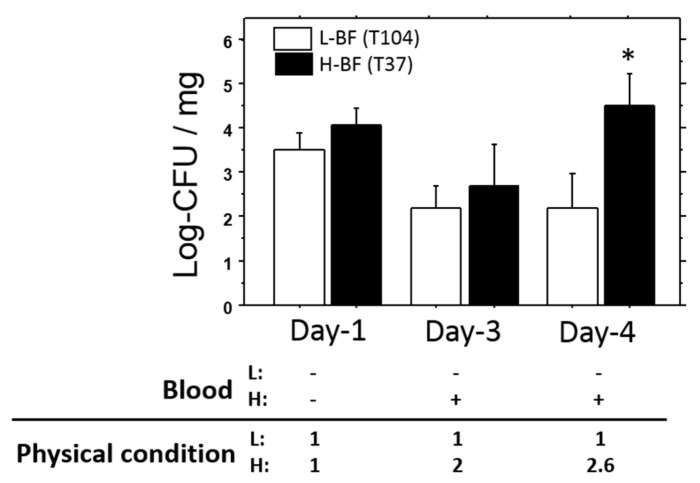
Persistence of MRSA in the liver and blood. * *p* < 0.05, -: negative, +: positive for CFU.

Persistence of viable bacteria in the liver was also examined for longer periods of time (1, 3, and 4 days). The representative data are shown in Figure 7. On day 1, the number of viable bacteria in T37 (H-BF) was slightly higher than that in T104 (L-BF), but was not significant. Both of the levels decreased on day 3. Subsequently, the level in T37 was elevated about two-fold on day 4. Its level was also significantly higher than that of T104 (*p* < 0.05). Bacteremia and deterioration of total condition were only found in T37 on days 3 and 4 of the study.

Diluted bacterial solution (1/2) was injected into mice, and the effects of the bacteria were examined on days 1, 3, and 4. Deterioration of physical condition and bacteremia were only found in H-BF (T37) on days 3 and 4. The viable bacterial count in the liver on day 4 was significantly higher in mice treated with T37 than in those treated with T104 (L-BF). Value: mean ± SEM.

## 3. Discussion

To date, there have been no reports of methods for the proper detection of MRSA biofilms within tissue samples. Once *S. aureus* enter the host’s body, their proteinaceous and non-proteinaceous adhesins mediate attachment to the extracellular matrix and cells [25]. They then produce extracellular materials and form biofilms. In order to detect biofilms formed within tissues, a staining method for polysaccharides was used, given that polysaccharides are present in large amounts in biofilms. EPS is known to have multi-functions, including the maintenance of biofilm structures, resistance to drugs, and evasion from the attacks of immune cells [7,8]. Staining of bacterial growth regions associated with necrotic foci formed inside the mice’s liver showed that while neutral mucopolysaccharides were negative in the matrix at the site of bacterial aggregation, the latter contained large amounts of acidic mucopolysaccharides. Meanwhile, with CV, a dye commonly used in biofilm research conducted in vitro, all components inside the tissue specimens were stained (unpublished data); therefore, CV staining could not be used for tissue studies. Acid mucopolysaccharides can also be found in mast cells, mesothelial cells, and goblet cells; therefore, caution is needed when searching for them in tissues. However, from a pathological viewpoint, the acid mucopolysaccharide staining method, especially ALB staining, which can be used for double staining, is simple, convenient, and effective for the identification of biofilm in MRSA-infected tissues. In addition to this, morphological observation and Gram staining should also be performed.

Enterotoxin, leukocidin, and toxic shock syndrome toxin (TSST) are known cytotoxic toxins produced by *S. aureus* [26,27]. We did not check the production of such toxins in the isolates. In the present study, bacterial culture solution at optical density of OD = 1.0 was considered to be a critical concentration to the mice. Some of the mice started to exhibit bacteremia 12 h after injection, and a deterioration of the general condition was found for some isolates. However, when 1/2-diluted bacterial solutions were used, the general condition was not affected for 24 h, even in the case of bacteria with the highest virulence. Therefore, bacterial solutions with an optical density of OD = 1.0 were used for the comparison of MRSA isolates in terms of tissue distribution and bacterial virulence.

One hour after injection of bacterial solution, most of the bacteria were distributed in hepatic, pulmonary, and splenic tissues; however, the bacterial count in the kidneys was lower and may have been dependent on phagocytosis systems. The time-course of bacterial tissue distribution showed that the bacterial counts in the liver remained unchanged, whereas those in the lungs and spleen decreased, suggesting that tissue distribution may be dependent upon bactericidal activity inside those tissues. This organ-specific distribution was also found in all of the isolates. Therefore, this may be a common phenomenon after MRSA enters the bloodstream. Meanwhile, the number of ALB-positive spots, which served as an indicator of putative biofilm formation, remained high only in the liver. In addition, the presence of biofilm was associated with the presence of bacteria inside Kupffer cells in the liver, and was accompanied by an increase in the size of clusters of intracellular bacteria, suggesting that the bacteria may have remained and/or proliferated intracellularly. In addition, the viable bacterial count in liver tissues at 24 h of the study also showed that a large number of bacteria were still present at that time. Phenol-soluble modulins (PMSs) are peptides involved in the maintenance and survival of endocytosed *S. aureus*. They are responsible for the destruction of the phagosome membrane, and are known to evade digestion [28].

Intracellular parasitism is a known phenomenon in certain bacteria, including *Listeria*, *Salmonella*, and *Mycobacterium tuberculosis*, which settle in host cells using various strategies against endosome-lysosomal and autophagy-based degradation systems [18,29,30,31]. A number of previous studies have shown that *S. aureus* may also survive intracellularly [16,32,33]. Various cells, such as epithelial cells [29,33,34], endothelial cells [35], and leukocytes including macrophages [17,21,36,37] and osteoblasts [38], have been reported to be invaded by persistent *S. aureus*. After invasion of a cell, *S. aureus* is believed to achieve survival by producing small colony variants (SCVs) [22,32,39]. On the other hand, when they are under stress due to pharmacological agents, bacteria have been reported to increase the appearance of persisters inside biofilms [11], and they favor conversion into SCV in host cells [12]. The bacterial distribution pattern inside liver tissues found in our study showed that the presence of ALB-positive substances in bacteria might help them to form biofilms and to convert into variants intracellularly.

In the present study, we examined biofilm activity in 173 clinical isolates. They showed a biologically normal distribution, from which the L-BF and H-BF isolates were selected. The presence of many of putative genes was checked as an essential factor for biofilm formation, but no difference was noted in the groups. It has been shown that regulatory genes, such as *icaADBC* [9] and *msaABCB* [40], influence biofilm development and virulence. Gene expression may change due to in vitro and in vivo circumstances. We plan to examine such gene expression in a future study.

The relationship between the biofilm-forming ability of MRSA and its bacterial virulence remains entirely unknown. Our previous study of foreign body infections using mice has shown that biofilm was crucial for bacterial survival in vivo [24]. However, it has been reported that biofilm is not a required condition for foreign body infection [41]. In the present study, when clinical isolates of MRSA were cultured under a comparable condition, each isolate showed a reproducible biofilm-forming ability. Comparison between the BF-L group and the BF-H group showed that the latter group induced a robust virulence in mice. In other words, the biofilm-forming ability observed in vitro may reflect an important influencing factor for bacterial survival inside a tissue cell; however, its intracellular kinetics still remain unknown. In fact, the virulence of some bacterial isolates in mice was not consistent with grouping. Hence, biofilm-forming ability is not the only determinant of virulence. However, identification of the biofilm-forming ability of MRSA in infected patients may serve as a reference for the determination of patient prognosis. Therefore, prospective studies will be needed in the future.

When bacterial virulence is induced, the condition of the infected organism is likely to shift to bacteremia, and tissue infection by bacteria is likely to be exacerbated. Therefore, given that the general condition was maintained within a normal range for all bacterial isolates inoculated as 1/2-diluted bacterial solutions, mice were injected with this amount of bacteria, and bacterial viability in the liver was compared at 24 h after injection. The findings showed that viable bacteria were 20-fold more numerous in the H-BF group than in the L-BF group. We furthermore checked their survival at longer periods up to 4 days. The number of viable cells decreased from 1 day to 3 days in both groups. However, the number of viable bacteria increased on day 4 in H-BF, but not in L-BF. This indicates that H-BF bacteria survived and started to proliferate inside the liver after a longer period of time. These results also support our hypothesis that biofilm-forming bacteria can persist intracellularly.

Biofilm-forming bacteria are known to have an enhanced resistance to the host’s immunity as well as antibiotics [6]. Biofilms are believed to develop drug resistance as a result of not only physical causes, such as a decreased permeability to drugs, but also bacterial conversion into persisters and/or dormant cells [6,7,42,43]. Our study suggested that after being phagocytosed, bacteria formed a biofilm intracellularly and evaded intracellular bactericidal mechanisms. Intracellular bacteria may slowly invade and extend into the hepatic tissue accompanied by extracellular biofilm, and form a large necrotic lesion after 10 days. Difficult strategies are required in order to overcome this, as antibiotics must achieve intracellular penetration. However, Lehar et al (2015) [44] recently proposed a novel method allowing for the elimination of *S. aureus* responsible for intracellular infection through antibody-antibiotic complexes. Development of effective treatment methods for the removal of intracellular *S. aureus* will be needed in the future.

Our study used a mouse model of bacteremia to show that the biofilm-forming ability of MRSA was important for maintaining the survival of bacteria in the host's body, and that MRSA was capable of intracellular persistence that could lead to chronic infection.

## 4. Experimental Sections

### 4.1. MRSA Isolates

We used one isolate of MRSA (OJ-1), which had been collected from the surface of an ulcerated wound and was kept at the Department of Plastic, Reconstructive, and Aesthetic Surgery, Fukuoka University Hospital. In addition, we also used a collection of 173 isolates of MRSA that had been kept at Fukuoka University Hospital, Department of Internal Medicine, Division of Oncology, Hematology and Infectious Diseases [45]. The bacterial isolates were thawed from the stocks and were incubated on Tryptic Soy Agar (TSA) (Becton, Dickinson and Company, Franklin Lakes, NJ, USA) containing 0.5% NaCl. When colonies formed, one colony was collected and scattered in 5 mL of Tryptic Soy Broth (TSB) (Becton, Dickinson and Company) in 12 mL plastic test tubes with screw caps (SARSTEDT, Tokyo, Japan), and bacterial culture was carried out at 37 °C [24]. Those that achieved a stable growth were cultured on agar again, and colonies formed were stored at 4 °C and were used in experiments within one month.

### 4.2. Extent of Biofilm Formation

Each MRSA isolate’s extent of biofilm formation was measured using the modified CV staining method [46]. Bacteria were harvested from TSA, suspended in test tubes containing 5 mL of TSB, and cultured at 37 °C for 24 h. Floating bacteria were discarded, and the biofilm that was adhering to the wall was washed with a phosphate buffer solution (PBS, pH 7.4). Then, 100% ethanol was added, and the samples were dried and subjected to CV staining for 10 minutes. After staining, the samples were washed in running water until the dye was completely eluted, and the dye that was attached to the surface of the test tubes was dissolved with 3 mL of 30% acetic acid. The absorbance of the dye solution (absorbance wavelength 570 nm) was measured using a spectrophotometer (GENESYS 10S VIS, Thermo Scientific, LMS, Tokyo, Japan). For all measurements conducted on clinical isolates from blood specimens, OJ-1 was used as a reference MRSA [24] and the CV values for all the tested isolates were calculated by considering that the CV value of OJ-1 was 1. The measurements of CV values were performed in triplicates and the mean values were considered the CV value for each isolate. On the basis of the extent of biofilm formation, 10 isolates with the highest biofilm-forming ability and 10 isolates with the lowest biofilm-forming ability were selected, and were classified as high-biofilm former (H-BF) and low-biofilm former (L-BF), respectively (Table 1).

### 4.3. Genotypic Characterization

In accordance with the previously reported method [47,48], genotypes *agr*, SCC*mec*, *sea*, *sec*, *sed*, *see*, *seg*, *she*, *sei*, *sej*, *sem*, *sen*, *tsst-1*, *icaA*, *icaD*, *hla*, *hlb*, *fnb*, *and fnbB* were amplified by PCR, and confirmation of the presence of fragments for these genes was carried out on the basis of band appearance and pattern using agarose gel electrophoresis.

### 4.4. Administration of MRSA Bacterial Solutions to Mice

The animal experiments that were carried out in this study had received prior approval from Fukuoka University Animal Center’s animal experiment approval committee (approval number: 1512884, 11 December 2015). One colony was harvested from the agar medium, suspended in TSB, and cultured at 37 °C. Bacterial culture in exponential phase was continued until the absorbance at a wavelength of 578 nm reached 1.0. A viable bacterial count at OD = 1, as assessed by CFU assay, showed no difference between isolates (about 2 × 10^4^ CFU/μL), and they were within 10% variation. Bacterial growth was then stopped by placing the TSB on ice, and the resulting bacterial solution was administered to animals in the subsequent experiments. In the experiments, the bacterial solution was used in its original concentration (×1) or diluted two-fold in TSB (1/2 dilution). Preliminary experiments have shown that when the bacterial solutions were used at a concentration of ×1, intravenous administration of bacteria of different species resulted in various systemic symptoms. Therefore, they were used for comparing the overall condition of the mice on the basis of differences in the MRSA isolates, as well as for determining the tissue distribution of bacteria. When used at a concentration of 1/2 dilution, neither of the bacterial solutions caused any considerable change in the mice's overall condition, and only a minimum number developed bacteremia; therefore, 1/2 dilution was used to study bacterial viability within tissues. Two hundred microliters of bacterial solution were administered to the caudal vein of female C57BL/6N mice between the ages of 6 to 10 weeks (Japan SLC Inc., Shizuoka, Japan) under anesthesia. At 1, 3, 6, 12 and 24 h and 3 and 4 days after administration of the bacterial solutions, the mice were killed by cervical dislocation and their organs (liver, lungs, spleen, and kidneys) were harvested and fixed in 10% neutral formalin.

### 4.5. Assessment of the Animals' Condition

A four-stage evaluation (1: normal, 2: hypoactive, 3: inactive, 4: dead) was used for the assessment of the animals' general condition after administration of bacteria. The evaluation of each bacterium was based on the mean value found in each mouse.

### 4.6. Histological Staining Method

The formalin-fixed tissues were washed with water, and paraffin blocks were prepared in a way that all 4 types of organs were contained in the same blocks. The paraffin blocks were cut into 4 μm-thick slices using a microtome, serial sections were attached onto a glass slide, and the necessary staining was performed. As general histological staining, hematoxylin-eosin staining (HE) and Gram staining were performed. As for acid mucopolysaccharide staining [49], Alcian blue staining (pH 2.5) (ALB), Toluidine blue staining (pH 2.5) (TB) and iron colloid staining (Fe colloid) were performed. As for neutral mucopolysaccharide staining, periodic acid-Schiff stain (PAS) was performed and immunostaining was carried out using an HRP-labeled anti-*S. aureus* antibody (ViroStat, Inc., Portland, ME, USA), and staining was developed using diamino benzidine after the reaction. For double staining with ALB, immunostaining was carried out after ALB staining.

### 4.7. Photographs of Tissue Samples for Analysis

The specimens that had been subjected to Gram staining and ALB staining were photographed using an optical microscope (Bio-Zero, KEYENCE Co., Osaka, Japan) on which observations were conducted with a 20× objective lens, and pictures were taken at 3 different locations where bacteria were present in the largest number inside the organs (image area: 134 × 10^3^ μm^2^, image resolution: 680 × 512 picts).

### 4.8. Morphometry

(1) Semi-quantitative evaluation of Gram-positive spots

Evaluation of tissue bacterial density: A four-level semi-quantitative evaluation method was carried out using Gram-stained specimens (0: absence of bacteria, 1: small amounts of bacteria, 2: moderate amounts of bacteria, 3: large amounts of bacteria). During the evaluations, image assessment of the photographs was carried out in blind, in reference to basic photographs that were considered as evaluation criteria. Reference photographs are Figure S2 for the liver, Figure S3 for the spleen, Figure S4 for the lung, and Figure S5 for the kidney. Mean values were used for the evaluations conducted on each bacterial isolate.

(2) Measurement of the number of Gram-positive spots

The photographs were imported into image analysis software (VH Analyzer ver2.60, KEYENCE Co., Kyoto, Japan), where a length calibration was performed, all Gram-positive spots with a size equal to or greater than 2 picts in the images were extracted, the number of positive spots was counted, and the area of each spot was measured. In addition, the number of spots with an area equal to or greater than 10 μm^2^ was also measured.

(3) Relevance of the area size of Gram-positive spots

To examine the bacterial distribution pattern of MRSA in hepatic tissues, spots in positive sites in Gram-staining photographs were numbered and their surface areas were measured. Their cumulative frequency distribution was analyzed using histograms.

(4) Measurement of the number of ALB-positive spots

The photographs of ALB-stained tissues were printed on A4-size photographic paper. ALB positive spots were detected macroscopically and counted. Three different people performed evaluations in blind, and the mean value was adopted as the value for the bacterial isolate.

### 4.9. Measurement of the Viable Bacterial Count within Liver Tissue

A bacterial solution with an optical density of OD = 1 (about 2 × 10^4^ CFU/μL) was diluted two fold in TSB, and 200 μL of the resulting solution was administered to mice intravenously through the caudal vein. Twenty-four hours later, blood samples were collected from the orbital cavity; liver tissues were also harvested and stored on ice. Blood samples and liver tissue samples, which had been processed with a homogenizer (Polytron PT 1200E: KINEMATICA AG, Luzern, Switzerland), were 10-times serially diluted and inoculated onto TSA. Bacterial culture was carried out at 37 °C for 24 h, and the number of colonies was counted as a colony-forming unit (CFU). For blood samples, the results were expressed as the number of colonies per μL, and for the liver tissue samples, the results were expressed as the number of colonies per mg of wet weight.

### 4.10. Statistical Analysis

Comparison of the two groups was carried out using the Student t-test and the chi-square test. Correlations between 2 factors were examined by linear regression analysis. The cumulative frequency distribution of the sizes of bacterial aggregates in the tissues was analyzed using histograms. *p* values <0.05 were considered significant.

## Figures and Tables

**Table 1:** Profiles of methicillin-resistant strains (MRSA) isolates. Summarized data of clinically isolated bacteria in the L-BF and H-BF groups, including biofilm-related genes and biofilm-forming ability (CV value in vitro). In addition, shown are each of the spot numbers from the photograph of liver tissue, including the Gram-stained spot number (G), more than 10 μm^2^ of Gram-stained spot number (G ≥ 10), and ALB-stained spot number 24 h after intravenous bacterial injection. Their physical condition classification is also indicated.

Group	Site (W/B)	Name	*agr*	SCC*mec*	*icaA*	*icaD*	*fnbA*	CV	Spot number per liver photo			Physical condition
									G	G>10	ALB	
**H-BF**	W	OJ-1	II	NT	+	+	+	1.000	30	5	7	3
**L-BF**	B	T87	I	II	+	+	+	0.087	21	1	1	1
	B	T104	II	II	+	+	+	0.105	34	4	3	1
	B	T96	II	II	+	+	+	0.117	24	4	2	1
	B	T101	II	II	+	+	+	0.124	32	6	2	3
	B	T109	II	II	+	+	+	0.124	28	4	5	1
	B	T90	II	II	+	+	+	0.125	16	1	2	1
	B	T153	II	II	+	+	+	0.128	20	1	3	1
	B	T126	II	II	+	+	+	0.140	51	7	3	1
	B	T2	I	NT	+	+	-	0.151	33	6	2	4
	B	T8	III	IV	+	+	+	0.156	40	2	3	1
**H-BF**	B	T146	II	II	+	+	+	0.980	31	6	3	4
	B	T125	II	II	+	+	+	1.078	34	6	4	4
	B	T34	II	II	+	+	+	1.107	25	3	8	1
	B	T166	II	II	+	+	+	1.112	26	3	3	4
	B	T32	II	II	+	-	+	1.116	36	9	4	1
	B	T144	II	II	+	+	+	1.138	35	6	6	1
	B	T41	I	NT	+	+	+	1.183	29	5	4	4
	B	T38	II	II	+	+	+	1.447	43	9	5	3
	B	T141	II	II	+	+	+	1.560	37	9	6	2
	B	T37	II	II	+	+	+	1.601	36	6	4	2

W: wound; B: blood

## Supplementary Materials

The following are available online at www.mdpi.com/2072-6651/8/7/198/s1, Table S1: Data of genes related to biofilm formation; Figure S1: A large infected necrotic lesion in the liver of a mouse 10 days after OJ-1 injection. After injection of OJ-1 ×1 solution, some mice survived and their general condition was fine 10 days after bacterial injection; however, large extracellular necrotic lesions were sometimes developed in the liver. The lesion was examined for biofilm formation. The result was similar to the lesion 24 h after injection (Figure 1); many MRSA colonies developed around the necrotic focus, and they were accompanied with acidic polysaccharides detected by Colloidal Fe, ALB, and TB staining, but were negative for neutral polysaccharides (PAS). The double staining of ALB + SA showed a biofilm matrix containing acidic mucopolysaccharides; Figure S2**:** Reference picture for grading of gram-positive MRSA in the liver. Each liver picture was evaluated using a set of reference pictures for the grading of gram-positive MRSA; Figure S3**:** Reference picture for grading of gram-positive MRSA in the spleen. Each spleen picture was evaluated using a set of reference pictures for the grading of gram-positive MRSA; Figure S4**:** Reference picture for grading of gram-positive MRSA in the lung. Each lung picture was evaluated using a set of reference pictures for the grading of gram-positive MRSA; Figure S5**:** Reference picture for grading of gram-positive MRSA in the kidney. Each kidney picture was evaluated using a set of reference pictures for the grading of gram-positive MRSA.

## Abbreviations

The following abbreviations are used in this manuscript:

MRSA	methicillin-resistant *S. aureu.*
PBP2’	peptidoglycan synthase.
SCC*mec*	staphylococcal cassette chromosome *mec.*
EPS	extracellular polysaccharides.
L-BF	low-biofilm formers.
H-BF	high-biofilm formers.
ALB	Alcian blue staining.
CV	Cristal violet.
TSST	toxic shock syndrome toxin.
PMSs	Phenol-soluble modulins.
