# Supplementary material for: Biofilm-Forming Methicillin-Resistant Staphylococcus aureus Survive in Kupffer Cells and Exhibit High Virulence in Mice
Source: Toxins (Basel). 2016 Jun 30;8(7):198. doi: 10.3390/toxins8070198 (PMC4963831; doi:10.3390/toxins8070198)
Supplement: Supplementary file 1 [file toxins-08-00198-s001.pdf]

Supplementary Materials: Biofilm-Forming Methicillin-Resistant *Staphylococcus aureus* Survive in Kupffer Cells and Exhibit High Virulence in Mice

Takuto Oyama, Motoyasu Miyazaki, Michinobu Yoshimura, Tohru Takata, Hiroyuki Ohjim and Shiro Jimi

Table S1. Data of genes related to biofilm formation.

Group	Name	sea	sec	sed	see	seg	seh	sei	sej	sem	sen	tsst-1	hla	hlb	fnbB
L-BF	87	-	+	-	-	+	-	+	-	+	+	+	+	+	-
L-BF	104	+	+	-	-	+	-	-	-	+	+	+	+	-	-
L-BF	96	+	+	-	-	-	-	+	-	+	+	+	+	-	-
L-BF	101	-	+	-	-	+	-	+	-	+	+	+	+	+	-
L-BF	109	+	+	-	-	+	-	+	-	+	+	+	+	+	-
L-BF	90	+	+	-	-	+	-	+	-	+	+	+	+	+	-
L-BF	153	-	+	-	-	+	-	+	-	+	+	+	+	+	-
L-BF	126	-	+	-	-	+	-	+	-	+	+	-	-	+	-
L-BF	2	-	-	-	-	-	-	-	-		+	-	-	-	-
L-BF	8	-	-	-	-	-	+	-	-	-	-	-	+	+	-
H-BF	146	-	+	-	-	+	-	+	-	+	+	+	+	-	-
H-BF	125	-	+	+	-	+	-	+	+	+	+	+	+	+	-
H-BF	34	-	+	-	-	+	-	+	-		+	+	+	-	-
H-BF	166	-	+	-	-	+	-	-	-	-	+	+	+	-	-
H-BF	32	-	+	-	-	+	-	+	-		-	+	-	-	-
H-BF	144	-	+	-	-	+	-	+	-	+	+	+	+	+	-
H-BF	41	-	-	-	-	-	-	-	-		-	-	+	-	+
H-BF	38	-	+	-	-	+	-	+	-	-	+	+	+	+	-
H-BF	141	-	+	-	-	+	-	+	-	+	+	+	+	+	-
H-BF	37	-	+	-	-	+	-	+	-	-	+	+	+	+	-

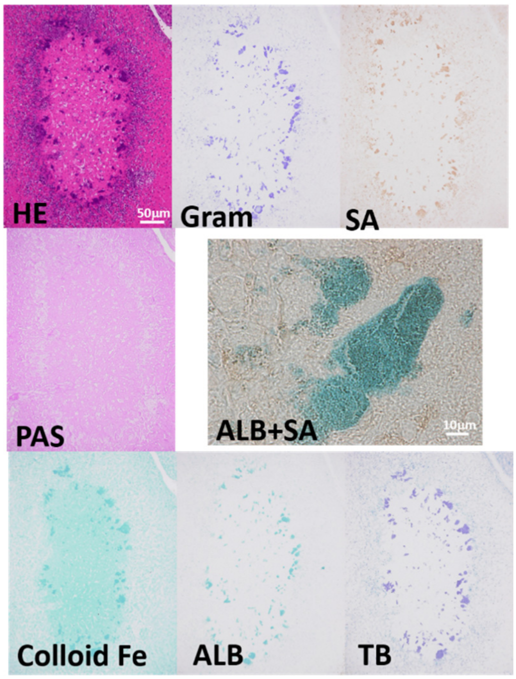

Figure S1. A large infected necrotic lesion in the liver of a mouse 10 days after OJ-1 injection. After injection of OJ-1 ×1 solution, some mice survived and their general condition was fine 10 days after bacterial injection; however, large extracellular necrotic lesions were sometimes developed in the liver. The lesion was examined for biofilm formation. The result was similar to the lesion 24 h after injection (Figure 1); many MRSA colonies developed around the necrotic focus, and they were accompanied with acidic polysaccharides detected by Colloidal Fe, ALB, and TB staining, but were negative for neutral polysaccharides (PAS). The double staining of ALB + SA showed a biofilm matrix containing acidic mucopolysaccharides.

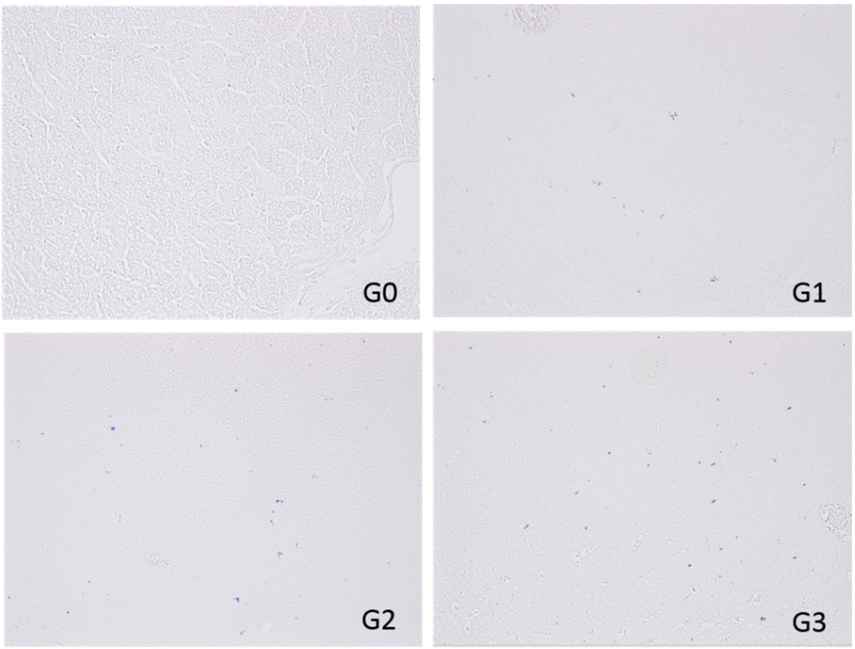

Figure S2. Reference picture for grading of gram-positive MRSA in the liver. Each liver picture was evaluated using a set of reference pictures for the grading of gram-positive MRSA. (330 × 440 μm^2)

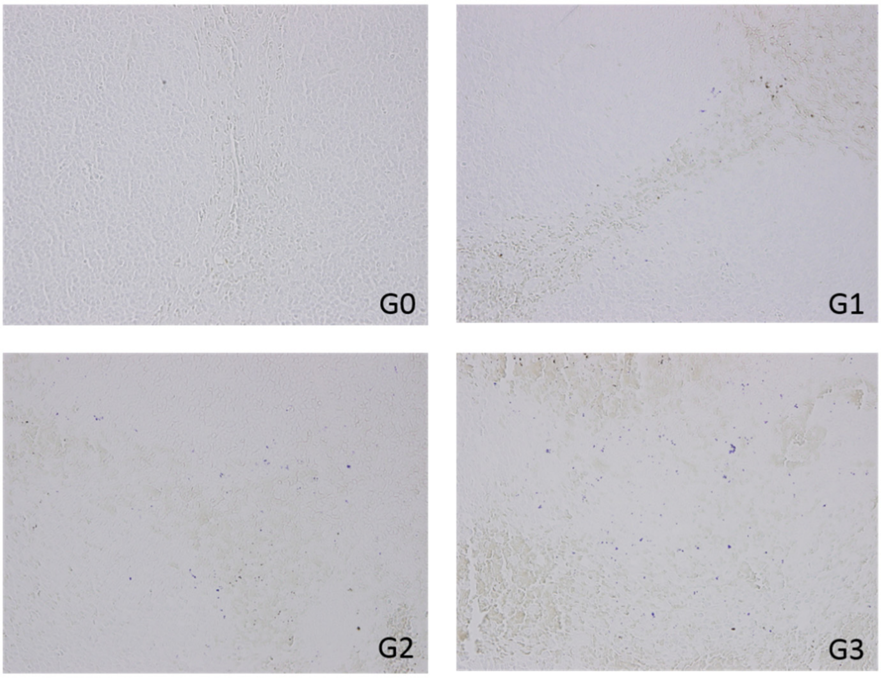

Figure S3. Reference picture for grading of gram-positive MRSA in the spleen. Each spleen picture was evaluated using a set of reference pictures for the grading of gram-positive MRSA. (330 × 440 μm^2)

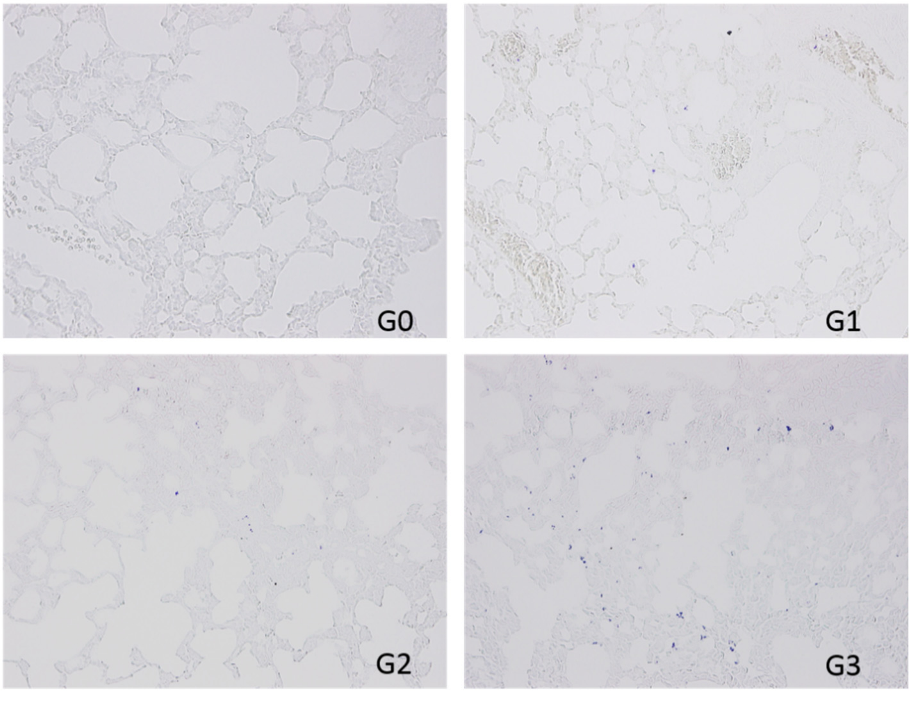

Figure S4. Reference picture for grading of gram-positive MRSA in the lung. Each lung picture was evaluated using a set of reference pictures for the grading of gram-positive MRSA. (330 × 440 μm^2)

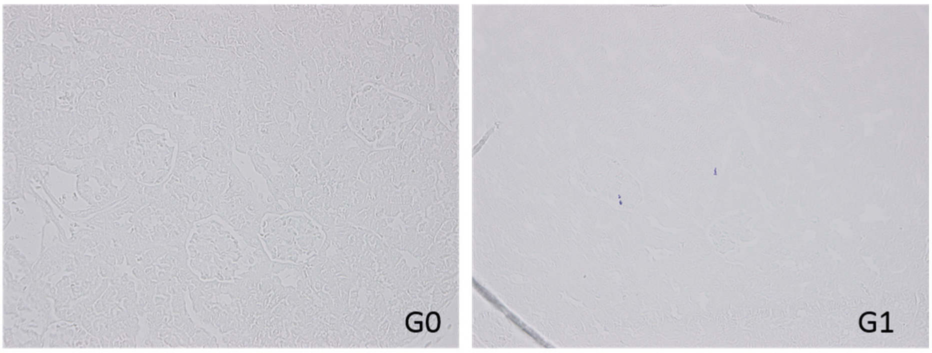

Figure S5. Reference picture for grading of gram-positive MRSA in the kidney. Each kidney picture was evaluated using a set of reference pictures for the grading of gram-positive MRSA. (330 × 440 μm^2)
